# Enhancing Access to Home Modification for Older Adults: Research Results of a National Survey of State Units on Aging

**DOI:** 10.1093/geroni/igaa057.160

**Published:** 2020-12-16

**Authors:** Julie Overton, Jon Pynoos, Emily Nabors, Damon Terzaghi, Bernard Steinman

**Affiliations:** 1 University of Southern California, Los Angeles, California, United States; 2 ADvancing States, Arlington, Virginia, United States; 3 University of Wyoming, Laramie, Wyoming, United States

## Abstract

With aging populations and cost constraints, state home and community-based care (HCBC) systems are increasingly being challenged to support older adults at home. Home modifications (HMs), changing the home environment to support health, safety and independence, has been found to be cost effective, improve quality of life, and help prevent falls. While the passage of the Older Americans Act in 1965 made State Units on Aging (SUAs) the designated state-level agencies responsible for developing and administering assistance to older adults, little is understood about the extent to which they prioritize, implement, and fund HM services. In the last decade, new developments have elevated HM on the public agenda, yet the creation of policies and programs depends on a solid knowledge base. Funded by the Administration for Community Living, the University of Southern California and ADVancing States conducted a survey of the 56 SUAs to ascertain HM efforts, targeting, Older Americans Act fund allocation for HMs, collaboration with other state entities, needs and challenges, legislation, and accomplishments. With a response rate of 91% SUAs (N = 51), the survey revealed: 88% of SUAs engage in HM efforts with most (61%) integrating HMs within HCBC and long term care programs; 74% work with their State Medicaid Office on including and delivering HMs via waivers; and the most pressing needs as funding and more HM providers. Analysis showed great variation of SUA involvement in HMs based on the state size, SUA location within the state government, and connections with other state agencies.

